# Dental Treatment Discontinuation for Financial Reasons Among Patients with Cancer: A Cross-Sectional Study with Non-Cancer Controls

**DOI:** 10.3390/jcm15020565

**Published:** 2026-01-10

**Authors:** Kyunghee Lee

**Affiliations:** Graduate School of Medical and Dental Sciences, Institute of Science Tokyo, Tokyo 1138519, Japan; kyungh.l.gm@gmail.com; Tel.: +81-3-5803-4025

**Keywords:** cancer survivors, financial toxicity, dental care utilization, treatment discontinuation, oral health-related quality of life (OHRQoL), Oral Health Impact Profile (OHIP-14)

## Abstract

**Background/Objectives**: The extent to which patients with cancer discontinue dental treatment for financial reasons remains unclear. This study compared the prevalence of financially driven dental treatment discontinuation between patients with cancer and without cancer (controls) and identified factors associated with discontinuation among patients with cancer. **Methods**: This cross-sectional, questionnaire-based survey conducted in November 2024 comprised 500 patients who had one of five common cancers in Japan and 500 non-cancer participants allocated to reflect the cancer group age distributions, sex, and household income. Only patients who received cancer treatment within the past 5 years and had a family dental clinic were included. Discontinuation was assessed from self-reported dental treatment cessation for economic reasons. Univariate analyses were employed for group comparisons (*p* < 0.05). **Results**: Dental treatment discontinuation for financial reasons occurred in 3.4% (95% CI 2.1–5.4%) of patients with cancer and 5.8% (95% CI 4.1–8.2%) of controls (*p* = 0.096). Among the patients with cancer, those who discontinued were younger (58.8 vs. 66.1 years, *p* = 0.010) and frequently reported physical or psychological barriers to dental care and discontinuation being financially driven (all *p* < 0.001). They also had poor oral health-related quality of life (Oral Health Impact Profile-14: 17.4 vs. 7.8, *p* < 0.001) and greater financial toxicity (Comprehensive Score for Financial Toxicity: 24.6 vs. 29.3, *p* = 0.001). **Conclusions**: Oral health-related quality of life was lower among participants reporting dental treatment discontinuation. Early identification of financial barriers and support may warrant further study.

## 1. Introduction

Cancer represents a substantial burden on global health; an estimated 19.3 million individuals were newly diagnosed worldwide in 2020 [1]. A wide range of oral complications can occur during cancer treatment, among which oral mucositis is particularly common [2]. Severe mucositis can cause intense pain and difficulty in eating, leading to not only diminished quality of life (QOL) and reduced physical strength but also disruptions in planned cancer treatment [3]. Thus, maintaining oral health is important for achieving optimal cancer treatment outcomes.

Managing oral complications requires dental care by oral health professionals prior to and during cancer treatment [4]. However, dental care involves financial costs, and the financial toxicity (FT) experienced by patients with cancer may influence their utilization of dental services [5,6,7]. FT is commonly used to describe the adverse effects of cancer-related medical expenses on patients’ QOL, reflecting both measurable financial strain and patient perceived financial distress [8,9]. In Japan, >60% of patients receiving chemotherapy report experiencing some degree of FT [10], and approximately 7.5% of bereaved family members report that recommended cancer treatments were discontinued or modified for financial reasons [11].

The extent to which FT affects dental care-seeking behavior among patients with cancer remains insufficiently understood. In particular, empirical data on the prevalence of dental treatment discontinuation for financial reasons and the factors associated with such discontinuation are limited. Few studies have characterized dental treatment discontinuation among patients with cancer, and comparisons to populations with cancer are lacking.

In this study, we compared the prevalence of dental treatment discontinuation for financial reasons between patients with cancer and those without cancer (controls) and identified factors associated with dental treatment discontinuation among patients with cancer.

## 2. Materials and Methods

### 2.1. Study Population and Study Design

This cross-sectional, questionnaire-based study comprised patients with cancer and a non-cancer comparison group allocated to reflect the cancer group age distribution categories, sex, and household income. The survey was conducted in November 2024 through Rakuten Insight, Inc. (Tokyo, Japan) [12], an Internet research company that maintains disease-specific respondent panels, including patients with cancer. Rakuten Insight has been widely used in epidemiological and clinical research employing web-based questionnaires, including studies involving patients with cancer [13,14,15]. Registered panelists voluntarily participate in multiple surveys conducted by the company and receive points or other incentives for participation. These incentives are not contingent on, nor intended to influence, the respondents’ answers. As of December 2022, the company had 21,105 registered patients with cancer, of whom 13,934 (66.0%) had one of the five most prevalent cancers in Japan (stomach, colorectal, lung, breast, or prostate cancer) based on data from the Cancer Information Service of the National Cancer Center, Japan [16]. Considering sample availability, the patients with these five cancers were targeted for recruitment.

### 2.2. Inclusion Criteria

As the primary focus of this study was dental treatment discontinuation for financial reasons, only individuals who had a family dental clinic were included.

For the cancer group, the inclusion criteria were (1) age ≥ 20 years; (2) had a family dental clinic; (3) diagnosis of a first primary cancer from among the five common cancer types; and (4) received cancer treatment (surgery, systemic therapy, immunotherapy, radiotherapy, interventional radiology, or palliative care) within the past 5 years.

For the non-cancer group, inclusion criteria were (1) age ≥ 20 years; (2) no cancer history; and (3) had a family dental clinic.

### 2.3. Exclusion Criteria

Exclusion criteria for the cancer group included receiving home medical care and diagnosis of multiple primary cancers. For the non-cancer group, receiving home medical care was the sole exclusion criterion.

### 2.4. Matching Procedure

To ensure comparability with the cancer group, the cancer group was surveyed first, and the age distribution categories, sex, and household income obtained from this group were used to guide participants allocations in the non-cancer group. Specifically, non-cancer-group participants were allocated to reflect the cancer group distributions across age categories (20 s, 30 s, 40 s, 50 s, 60 s, 70 s, and 80 s), sex, and household income categories. Household income categories were defined according to the questionnaire items (Supplementary Tables S1 and S2). This approach was intended to reduce socio-demographic imbalance between the groups at the recruitment stage, rather than through post hoc statistical matching. All the participants completed an online screening survey before accessing the main questionnaire. Only anonymized data were provided to the investigators, ensuring that no personally identifiable information was obtained. The target number of respondents was 500 per group (*n* = 1000 in total), with an upper limit of 100 respondents per cancer type in the cancer group.

### 2.5. Questionnaire Development

The questionnaire incorporated validated scales and items used in previous studies, along with original items developed to address the objectives of this study. The cancer group questionnaire consisted of 62 items across eight sections, and the non-cancer group questionnaire contained 25 items across five sections. The original items are presented in Supplementary Tables S1 and S2. They were designed with response categories tailored to the constructs being evaluated. The full versions of the previously validated scales are available in their respective published sources. Their response options and scoring procedures adhered to the original versions.

#### 2.5.1. A. Cancer Group Items

- A-1. Sociodemographic and clinical characteristics (13 items): Employment status and changes in employment [17], household income and income changes [17,18], household savings [10], type of health insurance, insurance copayment rate, type of family dental clinic [13], cancer stage, and performance status, which was originally developed by the Eastern Cooperative Oncology Group [19] and later published in Japanese by the Japan Clinical Oncology Group [20].
- A-2. Knowledge (7 items): Health knowledge items validated in previous studies [13], as well as added items regarding the Japanese High-Cost Medical Expense Benefit [21] and Medical Expense Deduction systems [22].
- A-3. Patient–dentist relationship (6 items): Items concerning explanations of treatment procedures and costs, based on prior research and clinical knowledge [13].
- A-4. Discontinuation of dental treatment for financial reasons (4 items): Financially driven discontinuation, timing, and type of dental treatment discontinued.
- A-5. Other types of treatment discontinuation (4 items): Discontinuation of dental treatment owing to physical or psychological reasons and discontinuation of cancer treatment for financial reasons.
- A-6. Oral health-related Quality of Life (OHRQoL) (14 items): The Oral Health Impact Profile (OHIP) is a widely used instrument for assessing OHRQoL, originally developed by Slade and Spencer [23], and its Japanese version has also been validated [24,25]. In this study, the short-form version, OHIP-14 [26], was used to evaluate OHRQoL. The participants were asked to respond to each item based on their experiences during the previous month, in accordance with the original instrument. The OHIP-14 uses a five-point response scale ranging from 0 to 4, resulting in a total score from 0 to 56; higher scores indicate poorer OHRQoL.
- A-7. FT (11 items): FT was assessed using the Japanese version of the Comprehensive Score for Financial Toxicity (COST), originally developed and validated by de Souza et al. [27,28]; the Japanese version was validated by Honda et al. [10]. The participants were asked to respond to each item based on their experiences during the previous 7 days, in accordance with the original instrument. The COST uses a five-point response scale ranging from 0 to 4, yielding a total score from 0 to 44; lower scores indicate greater FT.
- A-8. Life satisfaction (5 items): Life satisfaction was assessed using the Japanese version of the Satisfaction with Life Scale (SWLS), originally developed by Diener et al. [29]; the Japanese version was validated by Sumino [30]. The participants were asked to rate their agreement with each statement based on their current overall life situation, consistent with the original conceptual framework of the scale. The SWLS uses a seven-point response scale, yielding a total score ranging from 5 to 35; higher scores indicate greater life satisfaction.

#### 2.5.2. B. Non-Cancer Group Items

- B-1. Sociodemographic characteristics (10 items): Employment status, household savings, insurance type and copayment rate, and dental clinic type.
- B-2. Knowledge (4 items): Items that assessed general and oral health knowledge [13] and knowledge regarding the High-Cost Medical Expense Benefit [21] and Medical Expense Deduction systems [22].
- B-3. Patient–dentist relationship (5 items): Similar structure to the cancer group, developed based on earlier studies.
- B-4. Dental treatment discontinuation for financial reasons (3 items): Discontinuation of dental treatment for financial reasons within the past 5 years and type of treatment discontinued.
- B-5. Discontinuation for physical/psychological reasons (2 items): Discontinuation of dental treatment owing to physical or psychological reasons within the past 5 years. The 5-year recall window was selected to align with the eligibility criterion for the cancer group, which required participants to have received cancer treatment within the past 5 years; thus the timing of dental treatment discontinuation could be assessed over a similar period in both groups.

### 2.6. Variable Processing

The participants who answered “yes” to the question “Have you ever discontinued dental treatment for financial reasons?” (A-4-1 or B-4-1) comprised the discontinuation group, and those who answered “no” comprised the non-discontinuation group. For current employment status (A-1-1, B-1-1), free-text responses such as “pensioner” or “retired” were recoded as “unemployed,” and responses indicating non-regular employment were recoded as “part-time/temporary employment.” For employment changes (A-1-2), free-text comments indicating no difference before and after the cancer diagnosis, such as “I was already unemployed at diagnosis” or “I was not working,” were recoded as “no change.” For health insurance type (A-1-6), the responses describing “company health insurance” were interpreted as referring to Japan’s public health insurance category of “social insurance” and recoded accordingly. For the OHIP-14 (A-6-1 to A-6-14), reverse-scored items were processed according to the scoring method used in a previous study [26], and the total scores were recoded in their original 0 to 4 format. Items from the COST (A-7-1 to A-7-11) were recoded in their original 0 to 4 format [28]. For the SWLS (A-8-1 to A-8-5), reverse-scored items were processed according to the scoring method used in a previous study [29].

### 2.7. Statistical Analyses

Internal consistency of each scale (knowledge, patient–dentist relationship, OHIP-14, COST, SWLS) was assessed using Cronbach’s alpha. Dental treatment discontinuation for financial reasons was compared between the cancer and non-cancer groups using Fisher’s exact test (*p* < 0.05), and the absolute difference in discontinuation rates between the two groups was expressed as a risk difference with a 95% confidence interval. Sociodemographic characteristics were summarized descriptively, and group differences were evaluated using univariate analyses. Within the cancer group, comparisons between the discontinuation and non-discontinuation groups were also performed using univariate analyses. Fisher’s exact test was used for categorical variables, and *t*-tests or Mann–Whitney U tests were used for continuous variables after assessment of normality (*p* < 0.05). All analyses were performed using R software version 4.4.3 (R Foundation for Statistical Computing, Vienna, Austria) [31].

### 2.8. Consent to Participate

Survey invitations distributed by the research company described the study purpose, anonymity of responses, and voluntary participation. All respondents provided informed consent online before participating in the survey.

## 3. Results

### 3.1. Participants

A total of 2023 patients from the cancer panel participated in the preliminary screening, and 500 patients (100 for each of the five common cancers) completed the main cancer survey. For the non-cancer group, 500 participants were selected to reflect the cancer group age distribution categories, sex, and household income. Discontinuation of dental treatment for financial reasons was reported by 17 patients with cancer (3.4%, 95% CI 2.1–5.4%) and 29 participants without cancer (5.8%, 95% CI 4.1–8.2%); significant difference was not observed between the groups (*p* = 0.096). The absolute difference in discontinuation rates between the cancer and non-cancer groups was −2.4 percentage points (95% CI −6.1 to 1.3).

### 3.2. Internal Consistency of Scales

In the cancer group, Cronbach’s alpha values were 0.754 for the knowledge scale, 0.816 for the patient–dentist relationship scale, 0.965 for OHIP-14, 0.864 for COST, and 0.940 for SWLS. In the non-cancer group, Cronbach’s alpha was 0.735 for the knowledge scale and 0.906 for the patient–dentist relationship scale.

### 3.3. Participant Characteristics in the Cancer and Non-Cancer Groups

Participant characteristics for both groups are listed in Table 1. There were no significant differences in age, sex, or household income between the two groups. However, changes in household income differed significantly: 79.2% of patients with cancer reported no change in income, compared with 58.8% of the participants without cancer (*p* < 0.001). Utilization of the High-Cost Medical Expense Benefit system was more common among patients with cancer (77.0%) than among participants without cancer (24.4%) (*p* < 0.001). Utilization of the Medical Expense Deduction system was also higher among the patients with cancer than among the participants without cancer (73.4% vs. 47.4%, *p* < 0.001). Private health insurance coverage was more common among patients with cancer (57.2%) than among participants without cancer (22.8%) (*p* < 0.001). Patients with cancer had a significantly higher understanding of both the High-Cost Medical Expense Benefit system (3.2 vs. 2.9 points, *p* < 0.001) and Medical Expense Deduction system (3.3 vs. 3.1 points, *p* = 0.001).

### 3.4. Characteristics of the Discontinuation and Non-Discontinuation Groups Among Participants with Cancer

Characteristics of the discontinuation and non-discontinuation groups are presented in Table 2. The discontinuation group was significantly younger than the non-discontinuation group (58.8 ± 11.7 vs. 66.1 ± 9.2 years; *p* = 0.010). Cancer treatment status did not differ between groups; in both groups, >80% had completed active cancer treatment and were either attending regular follow-up visits or had completed all treatment and follow-up (*p* = 0.518). Discontinuation of dental treatment due to physical reasons (47.1% vs. 2.9%, *p* < 0.001) and psychological reasons (29.4% vs. 0.6%, *p* < 0.001) were markedly more common in the discontinuation group than in the non-discontinuation group. Financially driven discontinuation of cancer treatment was also significantly higher in the discontinuation group (17.6% vs. 0.6%, *p* < 0.001). Regarding FT, the discontinuation group had significantly lower COST scores than the non-discontinuation group (mean 24.6 ± 6.8 vs. 29.3 ± 4.9; *p* = 0.001), with medians of 26.0 and 30.0, respectively. The discontinuation group had poorer OHRQoL, as reflected by higher OHIP-14 scores than the non-discontinuation group (17.4 ± 11.9 vs. 7.8 ± 8.7; *p* < 0.001).

## 4. Discussion

This study examined the prevalence of dental treatment discontinuation for financial reasons among participants with cancer and explored associated factors by conducting comparisons between participants with and without cancer, as well as between dental treatment discontinuation and non-discontinuation groups within the cancer cohort. The primary between-group comparison showed no statistically significant difference in financially driven dental treatment discontinuation between participants with and without cancer. The results suggested that, in exploratory univariate analyses within the cancer group, participants who reported dental treatment discontinuation for financial reasons tended to be younger and to have poorer OHRQoL and greater financial toxicity than those who did not discontinue treatment. Given the very small size of the discontinuation group and the exclusive use of univariate analyses, these findings should be regarded as exploratory and hypothesis-generating rather than confirmatory. In addition, discontinuation was more frequently accompanied by physical or psychological barriers to dental care and financially driven discontinuation of cancer treatment. Overall, these findings suggest that dental care discontinuation may not be attributable to a single factor and may be associated with co-occurring economic, physical, and psychological difficulties.

As age, sex, and household income were aligned between the groups at the recruitment stage, the responses obtained from the participants with and without cancer were comparable. All scales demonstrated acceptable internal consistency [18,28,32,33,34]. The prevalence of discontinuation of dental treatment for financial reasons did not differ significantly between the cancer and non-cancer groups. Several factors may partly explain this observation. First, the participants with cancer reported higher utilization and understanding of the Japanese High-Cost Medical Expense Benefit [21] and Medical Expense Deduction systems [22], as well as greater use of private health insurance coverage, which may indirectly reduce the likelihood of dental treatment discontinuation by alleviating overall household financial strain, rather than through direct coverage of dental care [7]. Second, because the non-cancer group was allocated to reflect the age-category distribution of the cancer group, the age profiles of the two groups were broadly comparable, and both samples predominantly comprised older adults, who tend to have higher rates of multimorbidity, greater medical needs, and increased healthcare expenditures [35,36,37,38]. Consequently, economic and medical burdens may have been present among the participants without cancer as well, increasing their likelihood of discontinuing dental treatment and thereby reducing the between-group differences in discontinuation rates. Finally, a substantial proportion of respondents without cancer reported changes in household income, which may reflect broader economic disruption during the coronavirus disease 2019 pandemic in Japan, when approximately 25–30% of workers experienced adverse financial impacts [39], and income loss has been reported to be associated with declines in both physical and mental health [40]. The presence of such economically vulnerable individuals in the non-cancer group may have contributed to elevated financial strain in both groups, thereby narrowing the difference in discontinuation rates. Although the present study was not fully positioned to disentangle these overlapping influences, future studies should incorporate more detailed assessments of chronic disease status and income fluctuations to better isolate cancer-specific effects.

Within the cancer group, exploratory univariate analyses suggested that dental treatment discontinuation for financial reasons was more frequent among relatively younger patients (58.8 vs. 66.1 years), in line with prior reports indicating higher discontinuation rates among relatively younger dental patients in Japan [41] as well as evidence suggesting that younger age may be associated with greater FT among patients with cancer [7,42]. National survey data in Japan indicate that the prevalence of periodontal disease increases with age, surpassing 50% among individuals aged 55–59 years [43]. Discontinuation of dental care during an age when oral disease burden is rising may be associated with oral disease progression and potentially worsening cancer-related oral mucositis, which could be linked to disruptions in planned cancer treatment [3]. These findings underscore the importance of routinely identifying patients at high risk of discontinuation, particularly younger and economically vulnerable individuals, and connecting them to financial support systems at an early stage [7,44]. The observation that financially driven discontinuation of dental care was associated with physical and psychological burden and financially driven discontinuation of cancer treatment is consistent with findings from previous studies indicating that FT encompasses material, psychological, and behavioral burdens [7]. Patients who experience multiple overlapping burdens may be particularly susceptible to unstable care-seeking behavior. Patients often recognize the importance of discussing medical costs, although feel reluctant to initiate such discussions [5]; therefore, coordinated involvement of healthcare professionals, social workers, and insurers early in the treatment course may facilitate timely financial counseling and reduce undesirable treatment interruptions [5,7].

Although COST scores were significantly lower in the discontinuation group than in the non-discontinuation group, the corresponding mean scores (24.6 and 29.3) were classified as mild impact (Grade 1; COST 14–25) and no impact (Grade 0; COST ≥ 26), respectively, based on established grading criteria. Compared with previous studies of Japanese patients with cancer [10,18,42,45], FT in the present study appeared milder overall. This may be because many participants in the present study had already completed active cancer treatment or were only under periodic follow-up at the time of the survey. Since COST assesses financial burden over the “past 7 days,” FT may have been attenuated among individuals no longer undergoing active treatment. In contrast, many participants in previous studies were undergoing active therapy; the differences in the assessment timing may have contributed to the lower FT observed in this study. Therefore, COST in this study may reflect recent financial distress rather than peak or cumulative financial burden during active cancer treatment. Although the lack of longitudinal assessment precludes determining whether FT improves after treatment completion, the finding that FT remained associated with dental treatment discontinuation even after cancer treatment highlights the importance of addressing financial burden throughout survivorship.

OHIP-14 scores were significantly higher in the discontinuation group, suggesting persistently impaired OHRQoL even after completing cancer treatment, consistent with reports of reduced OHRQoL post-treatment among breast cancer survivors [46,47]. In contrast, OHIP-14 scores in the non-discontinuation group were comparable to those of community-dwelling older adults in Japan [34], suggesting that patients with cancer who continue dental care may maintain OHRQoL at levels similar to the general older population. Although longitudinal changes in OHRQoL were not assessed, the observation that lower OHRQoL was mainly reported among participants who discontinued dental treatment may suggest a possible association between dental care discontinuation and poorer OHRQoL after cancer therapy. These findings may warrant further study on barriers to maintaining dental care.

This study has a few limitations. First, the cross-sectional nature of the study does not allow causal relationships to be determined between dental treatment discontinuation for financial reasons, OHRQoL, and FT. Second, the small sample size of the discontinuation group limited statistical power and prevented multivariable analyses to identify independent associated factors, necessitating cautious interpretation of between-group comparisons. Third, oral health status and severity of dental disease were not assessed; therefore, the influence of these clinical factors on discontinuation could not be evaluated. Finally, because the study relied solely on questionnaire-based data and focused on the five common cancers, unmeasured factors and limited generalizability across cancer types should be acknowledged.

## 5. Conclusions

This study demonstrated that dental treatment discontinuation for financial reasons among patients with cancer was more common among relatively younger patients and tended to coincide with greater FT as well as physical and psychological burdens. Moreover, the discontinuation group exhibited poorer OHRQoL even after completing cancer treatment, whereas those who continued dental care maintained OHRQoL levels comparable to community-dwelling older adults. These findings suggest that dental treatment discontinuation may be associated with poorer oral health-related quality of life following cancer treatment. Early identification of financial barriers and the establishment of systems that support continuous dental care may help reduce avoidable dental treatment interruptions. As this was an exploratory, cross-sectional study, the findings should be interpreted as hypothesis-generating. Future studies using longitudinal designs are warranted to clarify the temporal relationships between financial burden, dental care continuity, and oral health outcomes, and to evaluate the effectiveness of targeted support interventions.

## Figures and Tables

**Table 1:** Characteristics of the cancer and non-cancer groups.

Item		Cancer Group (*n* = 500)	Non-Cancer Group (*n* = 500)	*p*		Type of Statistical Test
Age (years)	Mean (SD)	65.8 (9.4)	64.8 (9.6)	0.065		Mann–Whitney U test
	Median (IQR)	67.0 (60.0–73.0)	66.0 (60.0–72.0)			
Sex, *n* (%)	Male	360 (72.0)	360 (72.0)	1		Fisher’s exact test
	Female	140 (28.0)	140 (28.0)			
Current Employment Status, *n* (%)	Full-time employee	116 (23.2)	123 (24.6)	0.644		Fisher’s exact test
	Part-time/Temporary employee	79 (15.8)	90 (18.0)			
	Self-employed	31 (6.2)	38 (7.6)			
	On leave of absence	1 (0.2)	2 (0.4)			
	Unemployed (including full-time homemakers and students)	259 (51.8)	236 (47.2)			
	Other	14 (2.8)	11 (2.2)			
Household Savings, *n* (%)	<2 million JPY	100 (20.0)	104 (20.8)	0.48		Fisher’s exact test
	2 million JPY to <4 million JPY	64 (12.8)	81 (16.2)			
	4 million JPY to <6 million JPY	52 (10.4)	63 (12.6)			
	6 million JPY to <8 million JPY	31 (6.2)	28(5.6)			
	8 million JPY to <10 million JPY	35 (7.0)	33 (6.6)			
	10 million JPY to <15 million JPY	37(7.4)	37 (7.4)			
	≥15 million JPY	181 (36.2)	154 (30.8)			
Household Income, *n* (%)	<2 million JPY	59 (11.8)	59 (11.8)	1		Fisher’s exact test
	2 million JPY to <4 million JPY	178 (35.6)	178 (35.6)			
	4 million JPY to <6 million JPY	122 (24.4)	122 (24.4)			
	6 million JPY to <8 million JPY	60 (12.0)	60 (12.0)			
	8 million JPY to <10 million JPY	26 (5.2)	26 (5.2)			
	≥10 million JPY	55 (11.0)	55 (11.0)			
Change in Household Income, *n* (%)	Increased (≤2 million JPY)	8 (1.6)	10 (2.0)	<0.001	**	Fisher’s exact test
	Increased (≤1 million JPY to <2 million JPY)	5 (1.0)	17 (3.4)			
	Increased (<1 million JPY)	15 (3.0)	33 (6.6)			
	No change	396 (79.2)	294 (58.8)			
	Decreased (<1 million JPY)	36 (7.2)	59 (11.8)			
	Decreased (≤1 million JPY to <2 million JPY)	21 (4.2)	27 (5.4)			
	Decreased (≤2 million JPY)	19 (3.8)	60 (12.0)			
Type of Health Insurance, *n* (%)	National Health Insurance	224 (44.8)	224 (44.8)	0.782		Fisher’s exact test
	Social Insurance (e.g., Kyokai Kenpo, Union Insurance, Mutual Aid Association, etc.)	206 (41.2)	209 (41.8)			
	Medical Care System for the Older-Old	66 (13.2)	60 (12.0)			
	Other	0 (0.0)	0 (0.0)			
	Unknown	4 (0.8)	7 (1.4)			
Health Insurance Copayment Rate, *n* (%)	10%	49 (9.8)	38 (7.6)	0.131		Fisher’s exact test
	20%	117 (23.4)	145 (29.0)			
	30%	319 (63.8)	295 (59.0)			
	Other	2 (0.4)	2 (0.4)			
	Unknown	13 (2.6)	20 (4.0)			
Type of Dental Clinic, *n* (%)	Stand-alone dental clinic	480 (96.0)	475 (95.0)	0.222		Fisher’s exact test
	Clinic integrated with other medical departments	7 (1.4)	9 (1.8)			
	Dental department in a large general hospital	10 (2.0)	16 (3.2)			
	Other	3 (0.6)	0 (0.0)			
Use of Medical Systems/Insurance, *n* (%)	High-Cost Medical Expense Benefit system	385 (77.0)	122 (24.4)	<0.001	**	Fisher’s exact test
	Medical Expense Deduction system	367 (73.4)	237 (47.4)	<0.001	**	Fisher’s exact test
	Private health insurance	286 (57.2)	114 (22.8)	<0.001	**	Fisher’s exact test
Knowledge, mean (SD)	Healthy life expectancy	3.1 (0.7)	3.0 (0.8)	0.042	*	Mann–Whitney U test
	8020 movement	2.4 (1.1)	2.5 (1.1)	0.318		Mann–Whitney U test
	Understanding of High-Cost Medical Expense Benefit system	3.2 (0.8)	2.9 (0.8)	<0.001	**	Mann–Whitney U test
	Understanding of Medical Expense Deduction system	3.3 (0.7)	3.1 (0.7)	0.001	**	Mann–Whitney U test
Patient–Dentist Relationship, mean (SD)	Explanation of treatment content	3.2 (0.7)	3.2 (0.7)	0.344		Mann–Whitney U test
	Explanation of treatment costs	2.8 (0.7)	2.7 (0.8)	0.598		Mann–Whitney U test
	Consultation on treatment content	3.1 (0.6)	3.1 (0.7)	0.806		Mann–Whitney U test
	Consultation on treatment costs	2.9 (0.7)	2.9 (0.7)	0.556		Mann–Whitney U test
	Satisfaction	3.1 (0.6)	3.1 (0.6)	0.897		Mann–Whitney U test
Discontinuation of Dental Treatment for Financial Reasons t, *n* (%)	―	17 (3.4)	29 (5.8)	0.096		Fisher’s exact test
Details of Discontinued Treatment, *n* (%)	Oral care	3 (17.6)	—			Fisher’s exact test
	Fillings	3 (17.6)	4 (13.8)	1		Fisher’s exact test
	Crowns	3 (17.6)	6 (20.7)	1		Fisher’s exact test
	Dentures	1 (5.9)	7 (24.1)	0.226		Fisher’s exact test
	Periodontal treatment	5 (29.4)	4 (13.8)	0.258		Fisher’s exact test
	Endodontic treatment	3 (17.6)	1 (3.4)	0.135		Fisher’s exact test
	Cleaning/scaling	6 (35.3)	4 (13.8)	0.139		Fisher’s exact test
	Regular check-ups	4 (23.5)	8 (27.6)	1		Fisher’s exact test
	Whitening	0 (0.0)	3 (10.3)	0.286		Fisher’s exact test
	Implants	2 (11.8)	7 (24.1)	0.45		Fisher’s exact test
	Other private-pay treatment	0 (0.0)	1 (3.4)	1		Fisher’s exact test
	Other	0 (0.0)	3 (10.3)	0.286		Fisher’s exact test
Consultation at Discontinuation, *n* (%)	—	2 (11.8)	2 (6.9)	0.619		Fisher’s exact test

* *p* < 0.05, ** *p* < 0.01; IQR, interquartile range; SD, standard deviation.

**Table 2:** Characteristics of the discontinuation and non-discontinuation groups among participants with cancer.

Item		Discontinuation Group (*n* = 17)	Non-Discontinuation Group (*n* = 483)	*p*		Type of Statistical Test
Age (years)	Mean (SD)	58.8 (11.7)	66.1 (9.2)	0.01	*	Mann–Whitney U test
	Median (IQR)	58.0 (49.0–68.0)	67.0 (61.0–74.0)			
Sex, *n* (%)	Male	11 (64.7)	349 (72.3)	0.583		Fisher’s exact test
	Female	6 (35.3)	134 (27.1)			
Current Employment Status, *n* (%)	Full-time employee	6 (35.3)	110 (22.8)	0.473		Fisher’s exact test
	Part-time/Temporary employee	4 (23.5)	75 (15.5)			
	Self-employed	1 (5.9)	30 (6.2)			
	On leave of absence	0 (0.0)	1 (0.2)			
	Unemployed (including full-time homemaker and students)	6 (35.3)	253 (52.4)			
	Other	0 (0.0)	14 (2.9)			
Household Savings, *n* (%)	<2 million JPY	6 (35.3)	94 (19.5)	0.442		Fisher’s exact test
	2 million JPY to <4 million JPY	2 (11.8)	62 (12.8)			
	4 million JPY to <6 million JPY	0 (0.0)	52 (10.8)			
	6 million JPY to <8 million JPY	1 (5.9)	30 (6.2)			
	8 million JPY to <10 million JPY	2 (11.8)	33 (6.8)			
	10 million JPY to <15 million JPY	0 (0.0)	37 (7.7)			
	≥15 million JPY	6 (35.3)	175 (36.2)			
Household Income, *n* (%)	<2 million JPY	4 (23.5)	55 (11.4)	0.039	*	Fisher’s exact test
	2 million JPY to <4 million JPY	3 (17.6)	175 (36.2)			
	4 million JPY to <6 million JPY	2 (11.8)	120 (24.8)			
	6 million JPY to <8 million JPY	2 (11.8)	58 (12.0)			
	8 million JPY to <10 million JPY	3 (17.6)	23 (4.8)			
	≥10 million JPY	3 (17.6)	52 (10.8)			
Change in Household Income, *n* (%)	Increased (≤2 million JPY)	0 (0.0)	8 (1.7)	0.199		Fisher’s exact test
	Increased (≤1 million JPY to <2 million JPY)	1 (5.9)	4 (0.8)			
	Increased (<1 million JPY)	0 (0.0)	15 (3.1)			
	No change	12 (70.6)	384 (79.5)			
	Decreased (<1 million JPY)	1 (5.9)	35 (7.2)			
	Decreased (≤1 million JPY to <2 million JPY)	2 (11.8)	19(3.9)			
	Decreased (≤2 million JPY)	1 (5.9)	18 (3.7)			
Cancer Stage	0	4 (23.5)	76 (15.7)	0.831		Fisher’s exact test
	I	8 (47.1)	194 (40.2)			
	II	2 (11.8)	83 (17.2)			
	III	1 (5.9)	63 (13.0)			
	IV	1 (5.9)	20 (4.1)			
	Unknown	1 (5.9)	47 (9.7)			
Cancer Type, *n* (%)	Stomach cancer	2 (11.8)	98 (20.3)	0.544		Fisher’s exact test
	Colorectal cancer	2 (11.8)	98 (20.3)	0.544		Fisher’s exact test
	Lung cancer	5 (29.4)	95 (19.7)	0.353		Fisher’s exact test
	Breast cancer	6 (35.3)	94 (19.5)	0.123		Fisher’s exact test
	Prostate cancer	2 (11.8)	98 (20.3)	0.544		Fisher’s exact test
Family/Friend Cancer Experience	—	9 (52.9)	256 (53.0)	1		Fisher’s exact test
Cancer Treatment Status	Currently undergoing treatment	2 (11.8)	54 (11.2)	0.518		Fisher’s exact test
	Attending regular visits for next treatment	1 (5.9)	7 (1.4)			
	Treatment completed, attending regular visits for check-ups	10 (58.8)	289 (59.8)			
	All treatment and regular check-ups completed	4 (23.5)	129 (26.7)			
	Other	0 (0.0)	4 (0.8)			
Current Performance Status (PS), *n* (%)	1	14 (82.4)	420 (87.0)	0.528		Fisher’s exact test
	2	3 (17.6)	59 (12.2)			
	3	0 (0.0)	2 (0.4)			
	4	0 (0.0)	2 (0.4)			
Use of Medical Systems/Insurance, *n* (%)	High-Cost Medical Expense Benefit	14 (82.4)	371 (76.8)	0.773		Fisher’s exact test
	Medical Expense Deduction	15 (88.2)	352 (72.9)	0.262		Fisher’s exact test
	Private health insurance	10 (58.8)	276 (57.1)	1		Fisher’s exact test
Knowledge, mean (SD)	Healthy life expectancy	2.9 (0.7)	3.1 (0.7)	0.346		Mann–Whitney U test
	8020 movement	2.2 (1.1)	2.4(1.1)	0.323		Mann–Whitney U test
	Collaboration between dental and cancer care	1.6 (0.9)	1.6 (0.7)	0.574		Mann–Whitney U test
	Oral hygiene during cancer treatment	2.1 (0.7)	2.2 (1.0)	0.718		Mann–Whitney U test
	Oral complications	2.0 (0.9)	2.1 (1.0)	0.62		Mann–Whitney U test
	Understanding of High-Cost Medical Expense Benefit	2.9 (0.7)	3.2 (0.8)	0.163		Mann–Whitney U test
	Understanding of Medical Expense Deduction	3.0 (0.7)	3.3 (0.7)	0.089		Mann–Whitney U test
Patient–Dentist Relationship, mean (SD)	Explanation of treatment content	2.8 (1.0)	3.2 (0.6)	0.091		Mann–Whitney U test
	Explanation of treatment costs	2.5 (0.7)	2.8 (0.7)	0.079		Mann–Whitney U test
	Consultation on treatment content	2.8 (0.8)	3.1 (0.6)	0.055		Mann–Whitney U test
	Consultation on treatment costs	2.6 (0.7)	2.9 (0.7)	0.166		Mann–Whitney U test
	Dentist’s knowledge of cancer status	2.6 (1.0)	2.4 (1.1)	0.44		Mann–Whitney U test
	Satisfaction	2.9 (0.8)	3.1 (0.6)	0.238		Mann–Whitney U test
Treatment Discontinuation	Dental treatment discontinuation due to physical reasons	8 (47.1)	14 (2.9)	<0.001	**	Fisher’s exact test
	Dental treatment discontinuation due to psychological reasons	5 (29.4)	3 (0.6)	<0.001	**	Fisher’s exact test
	Cancer treatment discontinuation due to financial reasons	3 (17.6)	3 (0.6)	<0.001	**	Fisher’s exact test
	Cancer treatment discontinuation due to oral complications	1 (5.9)	3 (0.6)	0.13		Fisher’s exact test
OHIP-14	Mean (SD)	17.4 (11.9)	7.8 (8.7)	<0.001	**	Mann–Whitney U test
	Median (IQR)	14.0 (11.0–26.0)	5.0 (0.0–14.0)			
COST	Mean (SD)	24.6 (6.8)	29.3 (4.9)	0.001	**	Mann–Whitney U test
	Median (IQR)	26.0 (22.0–28.0)	30.0 (27.0–32.0)			
SWLS	Mean (SD)	17.5 (7.8)	20.1 (6.9)	0.095		Mann–Whitney U test
	Median (IQR)	18.0 (13.0–22.0)	20.0 (15.0–25.0)			

* *p* < 0.05, ** *p* < 0.01; COST, Comprehensive Score for Financial Toxicity; IQR, interquartile range; OHIP-14, Oral Health Impact Profile-14; SD, standard deviation; SWLS, Satisfaction with Life Scale.

## Data Availability

The data used in this study were obtained from a commercial survey panel provider (Rakuten Insight, Inc., Tokyo, Japan) and are subject to contractual restrictions on data sharing. Access to anonymized data may be granted to qualified investigators, upon reasonable request, from the corresponding author, subject to approval by the data provider and adherence to the relevant data use agreement.

## Supplementary Materials

The following supporting information can be downloaded at: https://www.mdpi.com/article/10.3390/jcm15020565/s1, Table S1: Original questionnaire items for the cancer group; Table S2: Original questionnaire items for the non-cancer group.
