# Supplementary material for: Dental Treatment Discontinuation for Financial Reasons Among Patients with Cancer: A Cross-Sectional Study with Non-Cancer Controls
Source: J Clin Med. 2026 Jan 10;15(2):565. doi: 10.3390/jcm15020565 (PMC12842033; doi:10.3390/jcm15020565)
Supplement: Supplementary file 1 [file jcm-15-00565-s001.zip › supplemenatry_tables_fv1.0.pdf]

Supplementary Table S1. Original Questionnaire Items for the Cancer Group

Item	Category	Question	Response Options
A-1 Sociodemographic Characteristics: Please select the most applicable option for the following items.			
A-1-1	Current Employment Status	What is your current employment status?	Full-time employee; Part-time/Temporary employee; Self-employed; On leave of absence; Unemployed (Including full-time homemaker, student); Other (Free text)
A-2 Knowledge: Please select the most applicable option for the following items.			
A-2-6	Understanding of High-Cost Medical Expense Benefit system	I know the meaning of the term "High-Cost Medical Expense Benefit system "	I know very well; I know somewhat; I don't know much; I don't know at all
A-2-7	Understanding of Medical Expense Deduction system	I know the meaning of the term "Medical Expense Deduction system "	I know very well; I know somewhat; I don't know much; I don't know at all
A-3 Patient–Dentist Relationship: Please select the most applicable option for the following items.			
A-3-1	Explanation of Treatment Content	My regular dentist thoroughly explains the content of my dental treatment.	Strongly agree (1); Somewhat agree (2); Somewhat disagree (3); Strongly disagree (4)
A-3-	Explanation of Treatment Costs	My regular dentist thoroughly explains the	Strongly agree (1); Somewhat agree (2); Somewhat disagree (3); Strongly disagree

2		costs of my dental treatment.	(4)
A-3-3	Consultation on Treatment Content	I can consult my regular dentist about anything concerning the content of my dental treatment.	Strongly agree (1); Somewhat agree (2); Somewhat disagree (3); Strongly disagree (4)
A-3-4	Consultation on Treatment Costs	I can consult my regular dentist about anything concerning the costs of my dental treatment.	Strongly agree (1); Somewhat agree (2); Somewhat disagree (3); Strongly disagree (4)
A-3-5	Dentist's Knowledge of Cancer	My regular dentist knows that I have/had cancer.	Strongly agree (1); Somewhat agree (2); Somewhat disagree (3); Strongly disagree (4)
A-4 Discontinuation of Dental Treatment for Financial Reasons: Please select the most applicable option for the following items.			
A-4-1	Discontinuation of Dental Treatment for Financial Reasons	I have experience changing or forgoing planned dental treatment due to financial reasons related to cancer treatment.	Yes; No

A-4-2	Timing of Discontinuation	(Only for those who answered "Yes" to A-4-1) When did you change or forgo the planned dental treatment? (Select all that apply)	Before cancer treatment; During cancer treatment; After completion of cancer treatment but during periodic follow-up visits; After completion of both cancer treatment and periodic follow-up; Other (Free text)
A-4-3	Discontinued Treatment Details	(Only for those who answered "Yes" to A-4-1) Which of the following dental treatments did you change or forgo? (Select all that apply)	Oral care during cancer treatment; Fillings (covered by insurance); Crowns (covered by insurance); Dentures (covered by insurance); Periodontal treatment (covered by insurance); Endodontic treatment (root canal treatment) (covered by insurance); Cleaning/scaling; Regular check-ups; Whitening; Implants; Other private-pay treatment (Free text); Other (Free text)
A-4-4	Consultation at Discontinuation	(Only for those who answered "Yes" to A-4-1) Were you able to consult your regular dentist when changing or forgoing dental treatment?	Fully able; Somewhat able; Not very able; Not able at all
A-5 Discontinuation Due to Other Reasons and Cancer Treatment Discontinuation: Please select the most applicable option for the following items.			

A-5-1	Discontinuation Owing to Physical Reasons	I have experience changing or forgoing planned dental treatment due to physical reasons related to cancer treatment.	Yes; No
A-5-2	Discontinuation Owing to Psychological Reasons	I have experience changing or forgoing planned dental treatment due to psychological reasons related to cancer treatment.	Yes; No
A-5-3	Discontinuation of Cancer Treatment for Reasons	I have experience changing or forgoing cancer treatment due to financial reasons.	Yes; No
A-5-4	Cancer Treatment Discontinuation Due to Oral Complications	I have experience changing or forgoing cancer treatment due to oral pain, inflammation, taste disorders, or other oral complications.	Yes; No

Supplementary Table S2. Original Questionnaire Items for the Non-Cancer Group

Item	Category	Question	Response Options
B-1 Sociodemographic Characteristics: Please select the most applicable option for the following items.			
B-1-1	Current Employment Status	What is your current employment status?	Full-time employee; Part-time/Temporary employee; Self-employed; On leave of absence; Unemployed (Including full-time homemaker, student); Other (Free text)
B-2 Knowledge: Please select the most applicable option for the following items.			
B-2-4	Understanding of Medical Expense Deduction system	I know the meaning of the term "Medical Expense Deduction system" (Iryōhi Kōjo).	I know very well; I know somewhat; I don't know much; I don't know at all
B-3 Patient–Dentist Relationship: Please select the most applicable option for the following items.			
B-3-1	Explanation of Treatment Content	My regular dentist thoroughly explains the content of my dental treatment.	Strongly agree (1); Somewhat agree (2); Somewhat disagree (3); Strongly disagree (4)
B-3-2	Explanation of Treatment Costs	My regular dentist thoroughly explains the costs of my dental treatment.	Strongly agree; Somewhat agree; Somewhat disagree; Strongly disagree
B-3-	Consultation on Treatment	I can consult my regular dentist about	Strongly agree; Somewhat agree; Somewhat disagree; Strongly disagree

3	Content	anything concerning the content of my dental treatment.	
B-3-4	Consultation on Treatment Costs	I can consult my regular dentist about anything concerning the costs of my dental treatment.	Strongly agree; Somewhat agree; Somewhat disagree; Strongly disagree
B-4 Discontinuation of Dental Treatment for Financial Reasons: Please select the most applicable option for the following items.			
B-4-1	Discontinuation of Dental Treatment for Financial Reasons	I have experience changing or forgoing planned dental treatment due to financial reasons in the past 5 years.	Yes; No
B-4-2	Discontinued Treatment Details	(Only for those who answered "Yes" to B-4-1) Which of the following dental treatments did you change or forgo? (Select all that apply)	Fillings (covered by insurance); Crowns (covered by insurance); Dentures (covered by insurance); Periodontal treatment (covered by insurance); Endodontic treatment (root canal treatment) (covered by insurance); Cleaning/scaling; Regular check-ups; Whitening; Implants; Other private-pay treatment (Free text); Other (Free text)

B-4-3	Consultation at Discontinuation	(Only for those who answered "Yes" to B-4-1) Were you able to consult your regular dentist when changing or forgoing dental treatment?	Fully able; Somewhat able; Not very able; Not able at all
B-5 Discontinuation Due to Other Reasons: Please select the most applicable option for the following items.			
B-5-1	Discontinuation Owing to Physical Reasons	I have experience changing or forgoing planned dental treatment due to physical reasons in the past 5 years.	Yes; No
B-5-2	Discontinuation Owing to Psychological Reasons	I have experience changing or forgoing planned dental treatment due to psychological reasons in the past 5 years.	Yes; No
